# Global, regional, and national lifetime risks of developing benign prostatic hyperplasia in men aged over 40: a population-based cross-sectional study from 1990 to 2021

**DOI:** 10.1186/s41182-025-00770-0

**Published:** 2025-07-14

**Authors:** Yongming Chen, Yuhao Li, Lingfeng Li, Miao Wang, Zhengtong Lv, Shengjie Liu, Huimin Hou, Shengfeng Wang, Ming Liu

**Affiliations:** 1 Beijing Hospital, National Center of Gerontology, Institute of Geriatric Medicine, Chinese Academy of Medical Sciences & Peking Union Medical College, Beijing, 100005 People’s Republic of China; 2 Department of Epidemiology and Biostatistics, School of Public Health, Key Laboratory of Epidemiology of Major Diseases, Peking University, Ministry of Education, Beijing, 100191 China; 3 Present Address: Department of Urology, Institute of Geriatric Medicine, Beijing Hospital, National Center of Gerontology, Chinese Academy of Medical Sciences, Beijing, 100005 People’s Republic of China; 4 Institute for Artificial Intelligence, Peking University, Beijing, China

**Keywords:** Global burden disease, Benign prostatic hyperplasia, Lifetime risk

## Abstract

**Background:**

Benign prostatic hyperplasia (BPH) is a prevalent condition among older men that significantly reduces quality of life. Despite its global impact, comprehensive estimates of lifetime risk remain limited. This study aims to quantify the lifetime risk of BPH at both global and regional levels using data from the Global Burden of Disease (GBD) 2021.

**Materials and methods:**

We estimated the lifetime risk of developing BPH in men aged over 40 using the “adjusted for multiple primaries (AMP)” method, derived from age-specific incidence rates provided by GBD. The analysis included data from 204 countries and regions covering the period from 1990 to 2021, stratified by age, region, and sociodemographic index (SDI).

**Results:**

In 2021, the global lifetime risk of BPH from age 40 to death was estimated at 27.29% (95% CI 27.26–27.31), with significant regional and socioeconomic inequalities. Eastern Europe exhibited the highest risk (37.57%), while Central Sub-Saharan Africa had the lowest (13.66%). When stratified by SDI, lifetime risk increased from 19.46% in low SDI regions to 31.51% in high-middle SDI regions but declined to 24.71% in high SDI settings. Across all regions, most cases were observed in individuals aged 50–70. Furthermore, between 1990 and 2021, the global lifetime risk of BPH showed a gradual increase. Projections indicate that this risk will remain stable over the next 30 years, with pronounced regional disparities expected to persist.

**Conclusions:**

This study offers a comprehensive assessment of the global lifetime risk of BPH, revealing significant regional variations and age-related trends. These findings underscore the need for targeted prevention and management strategies, particularly for high-risk regions and men aged 50–70, to reduce the global burden of BPH.

**Supplementary Information:**

The online version contains supplementary material available at 10.1186/s41182-025-00770-0.

## Introduction

Global population aging is accelerating. In 2016, approximately 617 million individuals were aged 65 years or older, accounting for 8.5% of the global population; this proportion is projected to rise to 17% by 2050 [1]. As a common condition among older men, benign prostatic hyperplasia (BPH) is expected to impose an increasingly substantial burden on healthcare systems worldwide. BPH is one of the most prevalent benign urological disorders [2]. Although it is non-malignant and does not directly affect survival, BPH often leads to lower urinary tract symptoms (LUTS) that significantly compromise the quality of life in aging male populations [3].

The global incidence of BPH has increased notably in recent decades—from 112.65 × million in 2019 [4] to 137.88 million in 2021 [5]—indicating a growing clinical and economic burden. Despite this trend, there remains a lack of systematic research evaluating the lifetime risk of BPH, particularly across different global regions. Lifetime risk provides an intuitive and cumulative measure of disease burden, estimating the probability of developing BPH over the remaining lifespan, assuming no competing causes of death [6]. This metric offers a meaningful framework for guiding long-term resource allocation, risk communication, and preventive strategies.

A previous meta-analysis by Shaun Wen Huey Lee et al. estimated the lifetime prevalence of BPH to be approximately 26.2% based on data from 25 countries [7]. However, that study relied primarily on literature synthesis and lacked standardized methodologies across regions. To date, no global, large-scale, and methodologically unified study has assessed the lifetime risk of BPH in a comprehensive manner.

The Global Burden of Disease (GBD) initiative provides a consistent and comparable source of epidemiological data across countries and over time [8]. Given that BPH is rare in men under 40 [9–11], the GBD dataset includes incidence estimates starting from age 40. Leveraging the GBD 2021 data, this study aims to estimate the lifetime risk of developing BPH across 204 countries and territories from 1990 to 2021, and to project future trends through 2050. The study provides evidence to support health policy decisions and prioritize regions in need of focused prevention and investment.

## Methods

### Data sources

This study utilized anonymized data from GBD 2021, led by the Institute for Health Metrics and Evaluation (IHME), a collaborative research initiative focused on estimating global population, fertility, incidence, and mortality rates [8]. The GBD study provides measurements of the impact of 371 diseases, 88 risk factors, and injuries across 204 countries and territories from 1990 to 2021 [12]. These 204 countries and territories are grouped into five social demographic index (SDI) levels and 21 GBD regions based on epidemiological similarity and geographic proximity.

We extracted data on age, sex, region, BPH-specific incidence and mortality rates, as well as total population and all-cause mortality rates for BPH patients from 1990 to 2021. The dataset encompasses all 21 GBD regions and 204 countries and territories. Notably, in the GBD framework, the mortality burden of BPH is reported as zero due to its chronic and non-lethal nature. Additionally, no BPH data are available for individuals under the age of 40. The SDI, a composite metric reflecting a country's overall development status, incorporates parameters such as per capita income, average years of education, and fertility rates among young women [12, 13]. SDI values range from 0 to 1 and are categorized into five levels: low (0–0.45), low-middle (0.45–0.61), middle (0.61–0.69), high-middle (0.69–0.81), and high (0.81–1.00) [14]. In addition to SDI-based stratification, the GBD framework also aggregates the 204 countries and territories into super regions based on geographic and epidemiological similarities. A summary of these GBD super regions and their constituent countries is provided in Supplementary Table 1.

The GBD modeling framework integrates various statistical methods, including Bayesian meta-regression through DisMod-MR 2.1, a compartmental model designed to ensure internal consistency between incidence, prevalence, remission, and mortality across diseases and locations. This modeling process incorporates data from multiple sources, adjusts for bias, and uses covariates to improve estimates where data are sparse. All estimates are generated with 1,000 draws from the posterior distribution to quantify uncertainty [15].

In GBD 2021, BPH was identified using the International Classification of Diseases (ICD) codes, including both the 10th Edition (ICD-10, coded as N40–N40.9) and the 9th Edition (ICD-9, coded as 600–600.91).

### Data analysis

#### Lifetime risks calculation

We calculated the lifetime risk of developing BPH by applying the adjusted for multiple primaries (AMP) method to age-specific incidence data from the GBD 2021 dataset. This method, previously described in detail [16, 17], estimates the cumulative probability of diagnosis over the remaining lifespan based on reported incidence rates and under the assumption of no competing mortality. The detailed calculation methods for lifetime risk are provided in the Supplementary Materials (pages 1–3). Moreover, the 95% confidence intervals (CIs) for AAPC estimates were obtained directly from the GBD 2021 data output, which provides uncertainty intervals derived from 1,000 posterior draws of the modeled disease burden. These intervals represent the 2.5th and 97.5th percentiles of the posterior distribution, accounting for data variability and model uncertainty.

#### Average annual percentage change

The temporal trend of lifetime risk was assessed using the average annual percentage change (AAPC) and its 95% confidence interval, calculated with the Joinpoint Trend Analysis software (command-line version 5.3.0). A positive AAPC indicates an increasing trend, whereas a negative AAPC signifies a declining trend.

#### Concentration index

We used the concentration index (CI) to quantify the inequality in disease burden across countries at different levels of development based on the SDI. The CI ranges from − 1 to 1, where a value less than 0 indicates that the disease burden is primarily concentrated in low SDI regions, while a value greater than 0 suggests that the burden is more prevalent in high SDI regions [18]. In addition, due to a significant increase in the risk of competing mortality, we excluded data from Rwanda (1994), Haiti (2010), and Somalia (2011) from the analysis.

#### Bootstrap-based frontier analysis

To assess the relationship between disease burden and socio-demographic development, we applied frontier analysis as a quantitative approach to estimate the potential achievable lifetime risk based on different levels of development measured by the SDI. Similarly, data from Rwanda (1994), Haiti (2010), and Somalia (2011) were also excluded. A detailed description of this method is provided in the Supplementary Materials (pages 3–4).

#### ARIMA forecasted model

We employed the AutoRegressive Integrated Moving Average (ARIMA) model [19, 20], a widely used time series forecasting method, to predict future trends in the lifetime risk of developing and dying from the disease under study. ARIMA combines three components: AutoRegression (AR), which models the dependency between an observation and a specified number of lagged observations; Integration (I), which applies differencing to make the series stationary; and Moving Average (MA), which captures the relationship between an observation and past forecast errors.

Using historical data from 1990 to 2019, we fitted ARIMA models to time series of annual disease risk values for each scenario. The best-fitting ARIMA model was selected using automated optimization, ensuring the parameters minimized the Akaike information criterion (AIC). Forecasts were generated for 2020–2050 years, and prediction intervals were calculated to quantify uncertainty. ARIMA model was implemented using the forecast package in R and results were visualized using ggplot2 R package, with actual and predicted values represented alongside 95% confidence intervals. The analysis also included a clear demarcation of historical and forecasted periods to highlight the model's applicability in projecting future disease burdens.

## Results

### Global lifetime risk of BPH

**Fig. 1:**
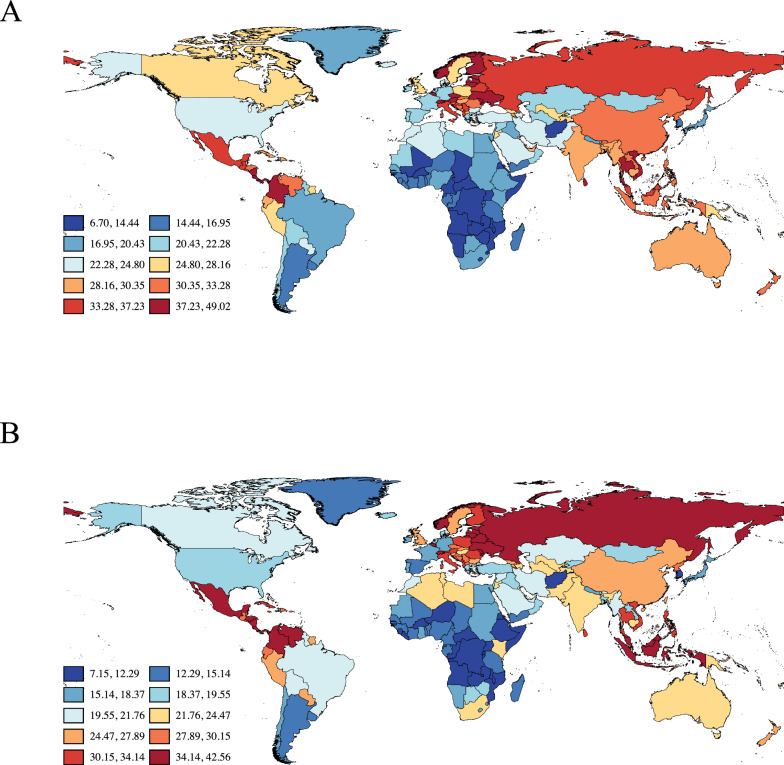
Global lifetime risk of developing BPH in males. **A**. Estimated lifetime risk of BPH from age 40 to death in 2021. **B**. Estimated lifetime risk of BPH from age 40 to death in 1990. Countries are color-coded by risk intervals (%), with darker red indicating higher lifetime risk and darker blue indicating lower risk. Estimates were derived using the AMP method applied to GBD 2021 age-specific incidence data. Risk values range from 6.70% to 49.02% in 2021 and from 7.15 to 42.56% in 1990

Using data from 2021, we calculated the lifetime risk of developing BPH from age 40 to death at global, regional, and national levels (Fig. 1A, Table 1, Supplementary Table 1). Globally, the lifetime risk of BPH from age 40 onward was 27.29% (95% CI 27.26–27.31). Eastern Europe exhibited the highest lifetime risk of BPH at 37.57% (95% CI 37.45–37.69), followed by Central Latin America (36.43%), Southeast Asia (33.35%), and East Asia (31.66%). In contrast, the lowest risks were observed in African regions, averaging around 15%. These findings highlight substantial regional disparities in the lifetime risk of BPH. Compared to the data from 1990 (Fig. 1B), the global lifetime risk of BPH in 2021 exhibited a modest increase, with minimal changes in magnitude and a largely consistent overall pattern.

**Table 1:** Lifetime risks of developing BPH by ages in 2021

Regions	40 years to death	50 years to death	60 years to death	70 years to death	80 years to death
Global	27.29 (27.26–27.31)	26.23 (26.21–26.26)	20.57 (20.55–20.59)	8.20(8.19–8.21)	1.22(1.22–1.22)
High SDI regions	24.71 (24.64–24.78)	23.77 (23.71–23.84)	18.81 (18.76–18.86)	8.33(8.31–8.36)	1.58(1.57–1.59)
High-middle SDI regions	31.51 (31.45–31.57)	30.59 (30.53–30.65)	24.73 (24.68–24.77)	10.35(10.33–10.38)	1.61(1.60–1.62)
Middle SDI regions	30.61 (30.56–30.65)	29.59 (29.54–29.63)	23.68 (23.65–23.71)	9.68(9.66–9.69)	1.44(1.43–1.44)
Low-middle SDI regions	26.05 (26.00–26.09)	24.68 (24.64–24.73)	18.21 (18.18–18.24)	6.08(6.06–6.09)	0.65(0.65–0.66)
Low SDI regions	19.46 (19.40–19.51)	18.37 (18.31–18.42)	13.19 (13.15–13.24)	4.36(4.35–4.38)	0.43(0.43–0.44)
High-income North America	22.61 (22.51–22.72)	21.21 (21.11–21.30)	16.77 (16.70–16.84)	8.78(8.74–8.82)	2.45(2.43–2.47)
East Asia	31.66 (31.60–31.72)	31.30 (31.25–31.36)	27.95 (27.90–28.00)	14.75(14.72–14.77)	2.71(2.70–2.72)
Central Asia	24.90 (24.70–25.10)	23.52 (23.33–23.71)	17.67 (17.52–17.82)	6.39(6.31–6.48)	0.73(0.71–0.75)
South Asia	29.34 (29.29–29.39)	27.67 (27.62–27.72)	20.61 (20.57–20.64)	6.90(6.89–6.92)	0.77(0.76–0.77)
Southeast Asia	33.35 (33.26–33.43)	31.72 (31.64–31.80)	23.10 (23.04–23.16)	7.52(7.49–7.55)	0.89(0.88–0.90)
High-income Asia Pacific	17.59 (17.41–17.77)	17.48 (17.30–17.66)	13.76 (13.62–13.89)	5.46(5.41–5.52)	0.65(0.63–0.66)
Central Sub-Saharan Africa	13.66 (13.54–13.77)	12.77 (12.66–12.89)	8.52 (8.43–8.61)	2.60(2.55–2.65)	0.23(0.21–0.24)
Eastern Sub-Saharan Africa	15.57 (15.50–15.65)	14.68 (14.61–14.76)	10.03 (9.97–10.09)	3.08(3.05–3.11)	0.28(0.27–0.28)
Southern Sub-Saharan Africa	15.72 (15.60–15.84)	14.60 (14.48–14.72)	8.74 (8.66–8.82)	2.30(2.27–2.34)	0.19(0.18–0.20)
Western Sub-Saharan Africa	16.24 (16.15–16.32)	15.34 (15.26–15.42)	10.77 (10.71–10.84)	3.82(3.79–3.85)	0.47(0.46–0.48)
North Africa and Middle East	21.35 (21.27–21.43)	20.38 (20.30–20.46)	15.14 (15.07–15.20)	5.92(5.89–5.95)	0.83(0.82–0.84)
Eastern Europe	37.57 (37.45–37.69)	35.44 (35.33–35.55)	23.01 (22.94–23.07)	5.75(5.72–5.78)	0.55(0.55–0.56)
Central Europe	30.73 (30.55–30.90)	29.27 (29.11–29.43)	22.50 (22.39–22.61)	8.41(8.36–8.46)	1.08(1.06–1.09)
Western Europe	26.50 (26.36–26.64)	25.37 (25.25–25.50)	18.50 (18.42–18.59)	6.52(6.48–6.56)	0.57(0.56–0.58)
Central Latin America	36.43 (36.29–36.58)	35.62 (35.48–35.77)	25.97 (25.86–26.07)	7.26(7.21–7.30)	0.69(0.68–0.70)
Andean Latin America	26.43 (26.17–26.69)	25.77 (25.52–26.03)	18.88 (18.69–19.07)	6.61(6.52–6.69)	0.77(0.74–0.79)
Southern Latin America	17.12 (16.89–17.35)	16.44 (16.22–16.66)	12.57 (12.40–12.73)	5.33(5.24–5.41)	0.90(0.87–0.93)
Tropical Latin America	19.90 (19.78–20.01)	19.37 (19.26–19.49)	15.05 (14.96–15.14)	5.87(5.82–5.92)	0.75(0.73–0.76)
Caribbean	27.25 (26.98–27.53)	26.63 (26.36–26.90)	19.94 (19.73–20.14)	7.02(6.93–7.12)	0.86(0.84–0.89)
Australasia	29.01 (28.45–29.57)	28.52 (27.97–29.06)	23.99 (23.57–24.41)	10.46(10.24–10.68)	1.62(1.56–1.69)
Oceania	25.40 (24.91–25.89)	24.47 (23.99–24.95)	19.15 (18.75–19.54)	6.66(6.46–6.85)	0.70(0.64–0.76)

### Changes in lifetime risk of BPH from 1990 to 2021

**Fig. 2:**
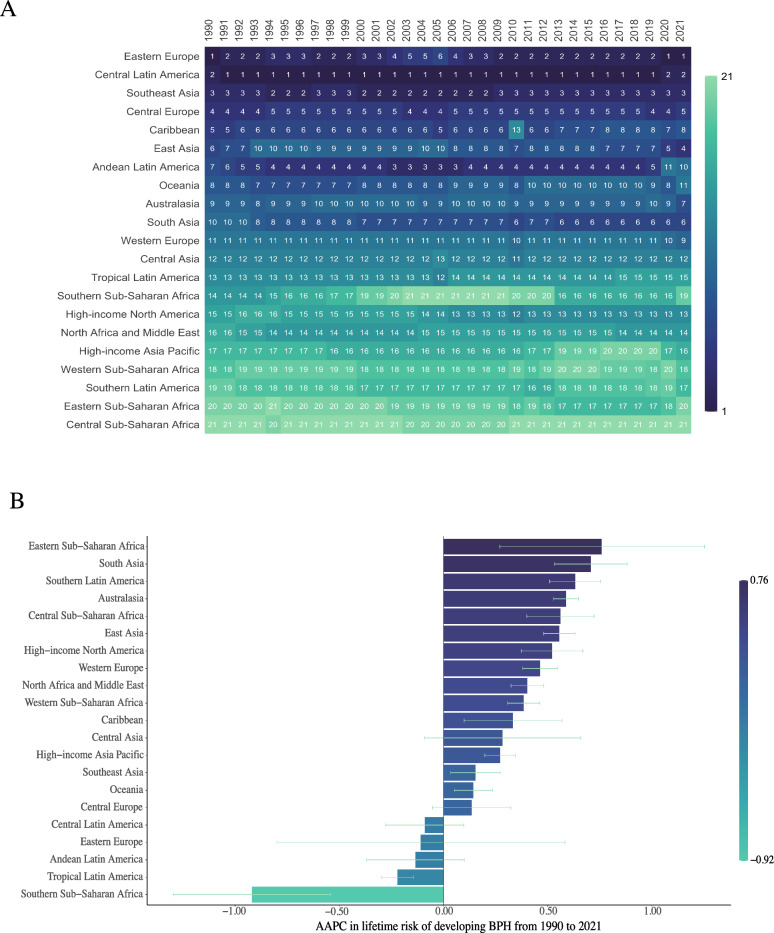
Regional trends in lifetime risk of developing BPH from 1990 to 2021. **A**. Heatmap showing the annual regional rankings of estimated lifetime risk of developing BPH. Rankings are based on modeled estimates using GBD 2021 age-specific incidence data and the AMP method. Each cell displays the rank (1 = highest risk; 21 = lowest risk), with darker colors indicating higher risk. **B**. AAPC in lifetime risk of BPH for each region during 1990–2021. Positive values indicate increasing trends; negative values reflect decreasing trends. Bars represent AAPC values, and error bars show the corresponding 95% confidence intervals

We further analyzed changes in the lifetime risk of BPH across regions from 1990 to 2021 (Fig. 2A). For most of the period (1991–2019), Central Latin America consistently had the highest lifetime risk globally. However, since 2020, Eastern Europe surpassed it as the region with the highest risk, while Southeast Asia remained among the top three throughout. In contrast, Africa exhibited the lowest lifetime risk globally, with Central Sub-Saharan Africa consistently ranking at the bottom.

To examine trends during this period, we calculated the AAPC for each region (Fig. 2B and Supplementary Table 2). Eastern Sub-Saharan Africa showed the most significant increase in lifetime risk, followed by South Asia and Southern Latin America. In contrast, Southern Sub-Saharan Africa experienced the most pronounced decline (− 0.9191%), far exceeding the reductions observed in other regions. Overall, the AAPC for lifetime risk in most regions ranged between 0 and 1%, indicating a general upward trend during this period.

### Socioeconomic inequalities in the lifetime risk of BPH

**Fig. 3:**
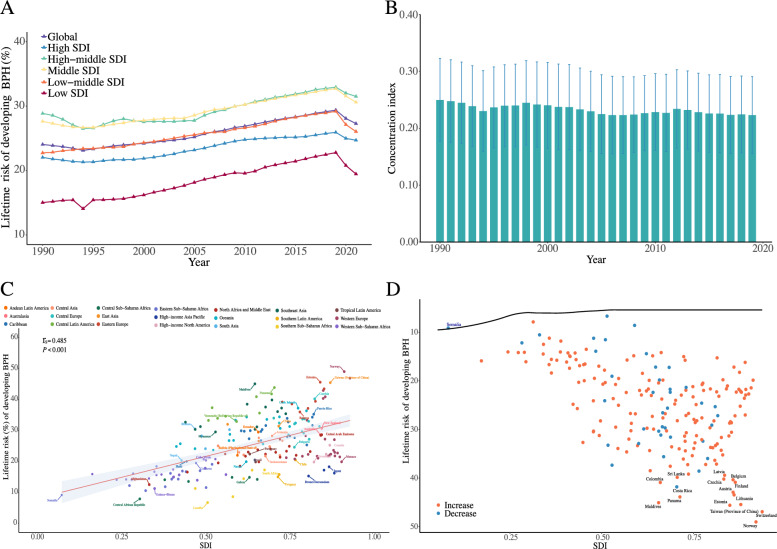
Global and regional trends in the lifetime risk of developing BPH, 1990–2021. **A**. Trends in the estimated lifetime risk of BPH from age 40 to death, stratified by SDI level. Risk estimates were derived using age-specific incidence data from GBD 2021 and calculated using the AMP method. **B**. Annual concentration index (CI) values from 1990 to 2021, indicating the degree of inequality in BPH burden across countries at different SDI levels. Higher CI values reflect greater concentration of risk in high-SDI countries. Error bars represent 95% confidence intervals. **C**. Cross-sectional correlation between SDI and lifetime risk (%) of BPH in 2021. Each point represents a country or region, colored by GBD region. A moderate positive correlation was observed (Spearman's *r*s = 0.485, p < 0.001). The red line denotes the fitted linear regression with 95% confidence band. **D**. Frontier analysis comparing each country’s observed lifetime risk in 2021 with the minimum achievable risk at its SDI level. The black curve represents the frontier (efficiency boundary). Countries above the frontier have higher-than-expected BPH risk, with the top 15 most divergent countries labeled. Red points indicate countries with increasing risk over time; blue points indicate decreasing risk

To explore the relationship between lifetime risk of BPH and socioeconomic development, we analyzed data based on the SDI (Table 1). From low SDI to high-middle SDI regions, the lifetime risk of BPH increased with higher SDI levels, rising from 19.46 to 31.51%. However, unexpectedly, regions with high SDI exhibited a decline in lifetime risk (24.71%). For instance, in developed regions such as Western Europe, the lifetime risk was only 26.50% (95% CI 26.36–26.64). Between 1990 and 2019, the lifetime risk of BPH exhibited a gradual but steady upward trend across all SDI regions, with no substantial alterations in the overall pattern (Fig. 3A and Supplementary Table 3).

To further assess the socioeconomic inequalities in the lifetime risk of BPH, we analyzed the CI from 1990 to 2021 (Fig. 3B and Supplementary Table 4). During the period, CI consistently remained above 0. Moreover, this inequality showed no significant improvement over the study period. Additionally, a moderate positive correlation was observed between the SDI and the lifetime risk of developing BPH (*r*_s_ = 0.485, *p* < 0.001), as shown in Fig. 3C and Supplementary Table 5. Higher SDI values were generally associated with an increased lifetime risk of BPH. The linear regression model highlights this trend, with the 95% confidence intervals reflecting potential variability in the relationship. These findings highlight the presence of socioeconomic inequality, with the burden of BPH being disproportionately concentrated in high SDI regions.

We conducted a frontier analysis to evaluate the gap between the actual lifetime risk of BPH in each country and the minimum achievable risk corresponding to its SDI level. Detailed results are presented in Supplementary Table 6 and 7. The results reveal that in 2021, BPH risk increased in most countries and Somalia was the only country that approached the minimum attainable risk level (Fig. 3D). Furthermore, the 15 countries that deviated the most from the curve, indicating the poorest risk control, were Norway, Switzerland, Estonia, Taiwan (Province of China), Maldives, Panama, Lithuania, Austria, Costa Rica, Colombia, Finland, Belgium, Czechia, Sri Lanka, and Latvia. Notably, most of these are high SDI countries. From a temporal perspective, though global SDI levels have improved from 1990 to 2021, most countries or regions have deviated further from the corresponding lowest risk of BPH over time (Supplementary Fig. 1).

### Strong association between lifetime risk of BPH and age

**Fig. 4:**
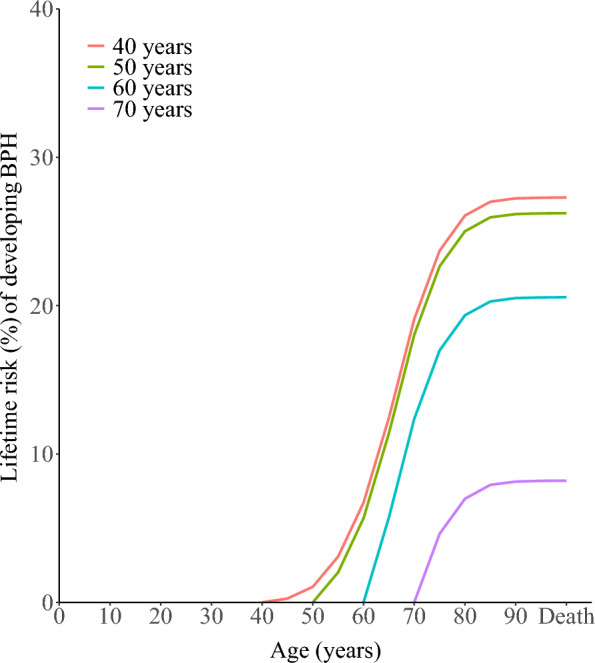
Lifetime risk of developing BPH by starting age in 2021. The figure displays the estimated cumulative lifetime risk (%) of developing BPH from various starting ages (40, 50, 60, and 70 years) to death, based on GBD 2021 age-specific incidence data and calculated using the AMP method. Each curve represents the trajectory of risk accumulation from the indicated starting age. The red, green, blue, and purple lines correspond to starting ages of 40, 50, 60, and 70 years, respectively

Globally, the risk of developing BPH from age 40 to death was 27.29% (95% CI 27.26–27.31), gradually decreasing with increasing age (Table 1). We visualized the relationship between lifetime risk and age on a global scale (Fig. 4 and Supplementary Table 8). The risks of “40 years to death” and “50 years to death” were very close (27.29% vs. 26.23%), indicating that the cumulative risk of developing BPH between ages 40 and 50 was only 1.03%. This suggests that most BPH cases occur after the age of 50.

Notably, the risk declined significantly after age 70, with the lifetime risk from “70 years to death” dropping to 8.20%. Beyond age 80, the increase in lifetime risk was minimal, with the “80 years to death” risk at only 1.20%.

### Forecasting future global lifetime risk of BPH

**Fig. 5:**
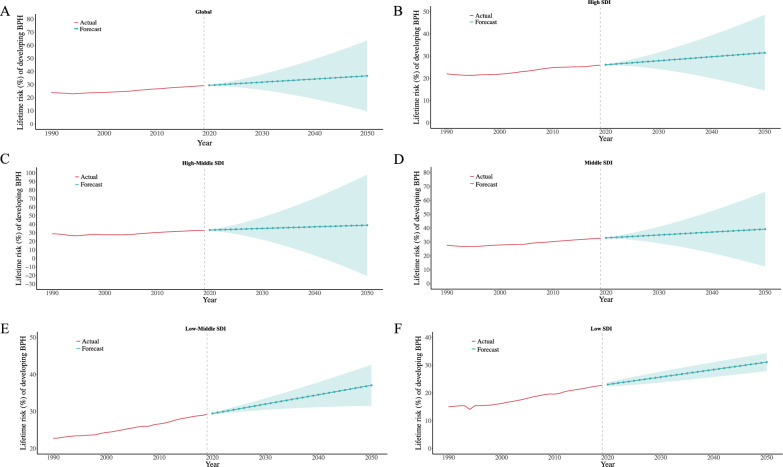
Projected trends in the lifetime risk of developing BPH from 1990 to 2050 based on ARIMA modeling across SDI levels. Panels A–F display the observed (1990–2019, red lines) and forecasted (2020–2050, blue lines) lifetime risk of developing BPH among males globally and across five Socio-demographic Index (SDI) categories. Forecasts were generated using an autoregressive integrated moving average (ARIMA) model based on historical trends. Shaded areas represent 95% confidence intervals of the projected estimates. **A**. Global; **B**. High SDI; **C**. High-middle SDI; **D**. Middle SDI; **E**. Low-middle SDI; **F**. Low SDI

Finally, we used an ARIMA model to predict the global lifetime risk of BPH in men over 40 years over the next 30 years. The analysis (Fig. 5A and Supplementary Table 9) indicates that while the risk has shown a gradual increase over the past decades, it is expected to stabilize in the coming years. The confidence intervals of the predictions reveal increasing uncertainty over time, reflecting potential variability in future trends. We further analyzed different SDI regions and found that in high, high-middle, and middle SDI regions, the lifetime risk of BPH is projected to maintain a relatively slow and steady increase over the next 30 years (Fig. 5B–D), closely aligning with the global trend. In contrast, low SDI regions exhibited a continuous increase in BPH lifetime risk from 1990 to 2019, and this upward trajectory is expected to persist over the next three decades (Fig. 5E and F). Moreover, the relatively narrow confidence intervals indicate a higher degree of certainty in these projections. These findings suggest that the future increase in BPH risk will be primarily driven by low SDI regions. These findings provide valuable insights for managing the global burden of BPH and developing targeted prevention strategies.

## Discussion

### Summary of key findings

This study provides a comprehensive analysis of the lifetime risk of BPH, with an estimated risk of one in four from age 40 to death. It also reveals substantial regional and socioeconomic disparities. Regions with higher levels of socioeconomic development generally exhibited a greater burden of BPH, although this trend plateaued in the most developed regions. Over the past three decades, the global lifetime risk of BPH showed a modest upward trend, with significant variation in changes across regions. Socioeconomic inequalities persisted throughout the study period, with no region achieving the minimum achievable risk level. Furthermore, age was strongly associated with BPH risk, with most cases occurring after 50 years and a marked decline observed in men aged over 70.

### Regional and socioeconomic disparities in BPH risk

To better understand the underlying drivers of the observed disparities, we examined geographic and socioeconomic variations in BPH lifetime risk. Eastern Europe had the highest lifetime risk, followed by Central Latin America and Southeast Asia. The elevated BPH risk in these regions may be attributed to a combination of aging populations, unhealthy lifestyles, and under-resourced healthcare systems. Recent evidence highlights that metabolic syndrome and its components—particularly insulin resistance, hypertension, and dyslipidemia—may promote prostate growth by altering hormonal and inflammatory pathways [21–23]. Obesity, both central and general, has also been associated with increased prostate volume and accelerated BPH progression, potentially mediated by adipokines and chronic low-grade inflammation [24–26]. Furthermore, insufficient physical activity not only exacerbates metabolic dysfunction but is independently linked to elevated BPH risk [27, 28]. Sedentary behavior and other lifestyle factors may further amplify these effects [29]. In parallel, recent multi-omics studies have identified immune-regulatory and inflammatory genes—such as *BTN3A2* and *C4A*—as contributors to BPH pathogenesis, implicating inherited differences in immune modulation and oxidative stress response as potential biological drivers of inter-individual and inter-regional variation [30]. These findings collectively underscore the dual importance of modifiable behavioral factors and intrinsic biological susceptibility in shaping global patterns of BPH burden. In contrast, Central Sub-Saharan Africa showed the lowest lifetime risk. This may be partly attributable to shorter life expectancy, limiting the period during which BPH can develop [31]. Moreover, lower diagnostic capacity and distinct lifestyle profiles—such as higher levels of physical activity—may also contribute to the lower observed risk [32].

Analysis of trends from 1990 to 2021 showed notable regional patterns. Central Latin America consistently had the highest risk until 2019. Since 2020, Eastern Europe surpassed it as the region with the highest lifetime risk. The reasons for this shift may include differences in healthcare access, lifestyle changes, and aging populations. East Asia showed fluctuating trends, with a decline followed by an increase in lifetime risk. Its ranking rose from 10 to 4th place, likely due to the region's accelerating aging population [33]. Through the analysis of AAPC, we observed distinct regional trends in BPH lifetime risk. Eastern Sub-Saharan Africa demonstrated the most pronounced increase in lifetime risk. Although the current burden of BPH in this region remains relatively low, continued economic development and shifting population demographics may lead to a rising risk and disease burden in the future [34]. Similarly, South Asia and Southern Latin America also showed significant increases in lifetime risk.

When stratified by SDI levels, a general positive correlation was observed between SDI and the lifetime risk of BPH. Regions with middle and high-middle SDI levels exhibited the highest risks, likely driven by longer life expectancy, as BPH tends to accumulate with age. Furthermore, lifestyle factors common in these regions, such as reduced physical activity, dietary changes, and increased exposure to environmental pollutants, may contribute to the higher prevalence of chronic conditions, including BPH [35–37]. Over the past three decades, the CI for BPH remained between 0.08 and 0.12, indicating that socioeconomic inequality in lifetime risk showed no improvement. However, in high SDI regions, the lifetime risk of BPH was lower and stable from 1990 to 2021, as seen in regions like Western Europe and High-income North America. This may be attributed to improved health awareness, stricter screening protocols, advanced diagnostic technologies, and healthier lifestyles. Interestingly, this trend contrasts with prostate cancer, which remains more prevalent in high SDI regions, indicating distinct relationships between SDI and these two diseases.

In contrast, low SDI regions likely underestimate BPH incidence due to limited diagnostic capacity and inadequate healthcare infrastructure. While SDI serves as a useful proxy for socioeconomic development, it has notable limitations. For example, SDI does not capture critical aspects of healthcare system quality, such as access to specialized services, efficiency, or affordability, which could influence the accuracy of disease burden estimates. Additionally, SDI averages may mask significant within-country disparities, especially in large or diverse nations, and its static nature fails to reflect dynamic changes in socioeconomic or healthcare conditions over time.

### Temporal patterns and forecasted trends

The ARIMA model analysis indicates that the lifetime risk of BPH has gradually increased over recent decades but is projected to stabilize in the next 30 years, potentially reflecting a global peak in BPH burden. This stabilization may be attributed to advancements in disease management, earlier diagnosis, and improved medical technologies, suggesting that current interventions are having a positive impact.

These findings underscore the need for tailored health interventions. High SDI regions should focus on addressing aging-related health challenges and ensuring effective long-term management. Meanwhile, low SDI regions require enhanced diagnostic capabilities and improved healthcare access.

Age group analysis revealed that the lifetime risk of developing BPH was 27.29% for men aged 40 to death and 26.23% for those aged 50 to death, with minimal difference between the two. In contrast, the risk dropped significantly to 8.20% for men aged 70 to death, representing a 69% decrease compared to the risk after age 50. This underscores the strong age dependence of BPH incidence, with a rapid accumulation of risk after midlife (50 years). Although the risk continues to accumulate with age, the rate of increase slows significantly in older age. These findings underscore the importance of focusing BPH prevention and management efforts on men aged 50–70, a critical window for intervention [38]. Strengthening risk screening and implementing early interventions during this period has the potential to significantly mitigate the disease burden.

In particular, low- and middle-income countries, where resource constraints may exacerbate rising incidence rates, warrant special attention. Although the overall trend appears to stabilize, rapidly aging regions such as East Asia remain areas of concern. Strengthening long-term management for aging populations, optimizing resource allocation, and promoting healthy lifestyles are essential strategies to mitigate the public health challenges posed by aging.

### Public health implications

Understanding the geographic and demographic distribution of lifetime BPH risk provides a valuable foundation for public health planning. Regions with high estimated risk—such as Eastern Europe and Central Latin America—may benefit from earlier screening initiatives and targeted preventive strategies, particularly for men aged 50–70, which represents a critical window for intervention. In areas where BPH risk is projected to rise, especially in low- and middle-SDI regions, expanding access to primary care and integrating BPH management into existing non-communicable disease (NCD) programs could enhance service delivery and continuity of care.

Beyond early detection, addressing disparities in treatment accessibility is equally important. Current therapeutic options for BPH include α-blockers, 5α-reductase inhibitors, combination pharmacotherapy, and minimally invasive surgical interventions [39, 40]. Compared to medications, surgery tends to provide greater improvement in symptom relief [41]. However, access, affordability, and adherence vary substantially across countries due to differences in healthcare infrastructure and economic capacity. In resource-limited settings, restricted availability of medications and surgical options may lead to delayed or inadequate treatment. Tailored policy efforts are needed to ensure equitable access to guideline-recommended therapies and to incorporate BPH care into broader frameworks for managing aging-related conditions.

The lifetime risk estimates presented in this study offer a practical reference for future resource allocation, including investment in diagnostic infrastructure, clinical services, and long-term care planning. As the global population continues to age, proactive expansion of both preventive and therapeutic services will be essential to mitigate the growing burden of BPH.

### Strengths and limitations

This study has several notable strengths. First, it provides a comprehensive evaluation of the lifetime risk of BPH in men by aggregating data from multiple regions and countries worldwide. The large-scale integration of data ensures broad representativeness, offering valuable insights for global BPH epidemiology. Additionally, the inclusion of diverse continents highlights significant differences in BPH risk across regions, serving as an important foundation for public health interventions.

Second, the detailed analysis of regional variations in BPH lifetime risk emphasizes the global disparities in disease burden. This approach identifies high-risk regions, such as Eastern Europe and Central Latin America, as well as low-risk regions, such as Sub-Saharan Africa, providing crucial guidance for region-specific prevention and management strategies.

Third, by examining trends in BPH lifetime risk from 1990 to 2021 across different regions, the study sheds light on the dynamic changes in disease burden over time. This temporal perspective is key to understanding the factors influencing BPH prevalence and highlights the evolving nature of its burden in various regions.

However, the study also has certain limitations. First, like all GBD studies, it is subject to general constraints [12, 31, 42, 43]. Despite covering a wide geographic scope, data limitations or incompleteness in resource-limited countries, particularly low-income nations, may affect the accuracy of the findings. Second, while this study integrates BPH data from diverse regions, heterogeneity in data collection methods and standards across regions may influence the precision of lifetime risk estimates. Third, although this study highlights regional differences and temporal trends in BPH lifetime risk, it does not fully explore the potential factors driving these risks, such as dietary habits, genetic predisposition, environmental pollution, and lifestyle changes. Future research should focus on more granular analyses to elucidate how these factors contribute to BPH risk across different regions and populations.

## Conclusion

Our study provides global, regional, and country-specific estimates of lifetime risk for BPH using GBD 2021 data. In 2021, one in four men globally were expected to develop BPH, with the highest risk in Eastern Europe and the lowest in Central Sub-Saharan Africa. Most cases occurred between ages 50 and 70, underscoring the importance of targeted prevention and management during this critical period. These findings highlight the need for age- and region-specific strategies to reduce the burden of BPH globally.

## Supplementary Information

Additional file 1.

Additional file 2.

## Data Availability

No datasets were generated or analyzed during the current study.
