# Supplementary material for: Global, regional, and national lifetime risks of developing benign prostatic hyperplasia in men aged over 40: a population-based cross-sectional study from 1990 to 2021
Source: Trop Med Health. 2025 Jul 14;53:93. doi: 10.1186/s41182-025-00770-0 (PMC12257823; doi:10.1186/s41182-025-00770-0)
Supplement: Supplementary file 1 — Additional file 1. [file 41182_2025_770_MOESM1_ESM.docx]

Methods of calculating lifetime risk of developing BPH

The lifetime risk of developing BPH, as discussed in this study, estimates the probability of being diagnosed with BPH within the population. In contrast, it also refers to the likelihood of actually developing BPH in the population. Commonly used methods for such estimations include the cumulative rate, cumulative risk, and life table approaches.

1. Cumulative rate

$$Cumulative rate=\sum_{i=1}^{A} W_{i}R_{i}$$

A represents the age band for cumulative rate calculations. Wi denotes the width of the ii-th age interval, and RiRi​ represents the age-specific incidence rate.

1. Cumulative risk

The cumulative risk can be calculated by the formular:

$$Cumulate risk=1-e^{-cumulative rate}$$

1. The “adjusted for multiple primaries (AMP)” method

The AMP method has been introduced in detail in previously study ^[1,2]^. Lifetime risk was calculated by five-year age groups using all-cause mortality and BPH incidence and mortality data:

$$S=\int_{0}^{\infty} \lambda_{c}\left( a \right)S_{0}\left( a \right)\mathrm{da}=\sum_{i=1}^{f} \frac{R_{i}}{R_{i}+M_{i}-D_{i}}\hat{S}_{0}^{*}\left( a_{i} \right)\times\left( 1-exp\left( -\frac{w_{i}}{N_{i}}\left( R_{i}+M_{i}-D_{i} \right) \right) \right)$$

For age group i:

S: Probability of being diagnosed with BPH;

Mi​: Annual number of deaths (all-cause mortality);

Di​: Annual number of BPH-related deaths;

Ri​: Annual number of BPH cases;

Ni​: Population size;

λc​: BPH incidence rate;

S ̂_0^* (ai ): Probability of being alive and BPH-free at age ai;

Wi​: Width of the ith age group;

where the i^th^ interval is from a_i_ to a_i+1_,

w_i=_ (a_i+1_-a_i_)

$$\hat{S}_{0}^{*}\left( a_{i} \right)=exp (-\sum_{j=1}^{i-1} \frac{R_{j}{+ (M}_{j}-D_{j})}{N_{j}})$$

In practice, BPH incidence and mortality data are usually presented in the form of frequency table for calculation, and the final age band is usually 85 years old or above (e.g.0-4,5-4,10-10, …, 80-84, 85+). For the final age band (e.g. 85+), the integral for the final age band evaluates to the following:

$$\frac{R_{f}}{R_{f}+M_{f}-D_{f}}S_{0}\left( a_{f} \right)$$

The derivation of variance to the following:

let： $\lambda_{c}=\frac{R_{i}}{R_{i}+M_{i}-D_{i}},\hat{S}_{0}^{*}=\hat{S}_{0}^{*}\left( a_{i} \right),S_{x}=\left( 1-\exp\left( -\frac{w_{i}}{N_{i}}\left( R_{i}+M_{i}-D_{i} \right) \right) \right)$ then

$Var\left( S \right)=\sum_{i=1}^{f} Var\left( \lambda_{c}\hat{S}_{0}^{*}S_{x} \right)$=$\sum_{i=1}^{f} \left[ E\left( \lambda_{c}^{2}\left( \hat{S}_{0}^{*} \right)^{2}S_{x}^{2} \right)-{E\left( \lambda_{c}\hat{S}_{0}^{*}S_{x} \right)}^{2} \right] (1)$

$$E\left( \lambda_{c}^{2}\left( \hat{S}_{0}^{*} \right)^{2}S_{x}^{2} \right)=E (\lambda_{c}^{2})E\left( \hat{S}_{0}^{*} \right)^{2}E\left( S_{x}^{2} \right)=\left( var\hat{(S}_{0}^{*})+{(E\hat{S}_{0}^{*})}^{2}][Var\left( S_{x} \right)+{E (S_{x})}^{2}]\} \right)$$

$$Var\left( S \right)=\sum_{i=1}^{f} \{[Var (\lambda_{c})+\left( E\lambda_{c} \right)^{2}][Var\left( \hat{S}_{0}^{*} \right)+\left( E\hat{S}_{0}^{*} \right)^{2}][Var\left( S_{x} \right)+\left( ES_{x} \right)^{2}-\left( E\lambda_{c} \right)^{2}\left( E\hat{S}_{0}^{*} \right)^{2}\left( ES_{x} \right)^{2}\}$$

Let:

$p_{ci}=\frac{R_{i}}{R_{i}+M_{i}-D_{i}},$ then

$$E\lambda_{c}=p_{ci}, \left( Binomial\& Poisson \right) (2)$$

$$Var\left( \lambda_{c} \right)=\left\{ \begin{aligned} \frac{p_{ci}\left( 1-p_{ci} \right)}{R_{i}+M_{i}-D_{i}}, \left( Binomial \right) \\ \frac{p_{ci}}{R_{i}+M_{i}-D_{i}}, \left( Poisson \right) \end{aligned} (3) \right.$$

$\log\left( \hat{S}_{0}^{*} \right)=-\sum_{j=1}^{i-1} \frac{R_{j}+M_{j}-D_{j}}{N_{j}}$, let $p_{oj}=\frac{R_{j}+M_{j}-D_{j}}{N_{j}},$ then

$$Var\left( log\hat{S}_{0}^{*} \right)=\sum_{j=1}^{i-1} Var\left( \frac{R_{j}+M_{j}-D_{j}}{N_{j}} \right)=\left\{ \begin{aligned} \sum_{j=1}^{i-1} \frac{p_{oj}\left( 1-p_{oj} \right)}{N_{j}}, (Binomial) \\ \sum_{j=1}^{i-1} \frac{p_{oj}}{N_{j}}, (Poisson) \end{aligned} \right.$$

According to delta method, if $\hat{\theta}\sim N\left( \theta,\sigma^{2} \right)$, then $f\left( \hat{\theta} \right)\sim N (f\left( \theta\right),{[f^{'}\left( \theta\right)]}^{2}\sigma^{2})$. Let $\hat{S}_{0}^{*}=e^{\hat{\theta}}, then$

$$E\left( \hat{S}_{0}^{*} \right)=\exp\left( -\sum_{j=1}^{i-1} p_{oj} \right), \left( Binomial \& Poisson \right) (4)$$

$$Var\left( \hat{S}_{0}^{*} \right)={(e^{\hat{\theta}})}^{2}Var\left( log\hat{S}_{0}^{*} \right)=\left( \hat{S}_{0}^{*} \right)^{2}Var\left( log\hat{S}_{0}^{*} \right)=\left\{ \begin{aligned} \left( \hat{S}_{0}^{*} \right)^{2}\times\sum_{j=1}^{i-1} \frac{p_{oj}\left( 1-p_{oj} \right)}{N_{j}}, \left( Binomial \right) \\ \left( \hat{S}_{0}^{*} \right)^{2}\times\sum_{j=1}^{i-1} \frac{p_{oj}}{N_{j}}, \left( Poisson \right) \end{aligned} \right. (5)$$

$S_{x}=\left( 1-\exp\left( -\frac{w_{i}}{N_{i}}\left( R_{i}+M_{i}-D_{i} \right) \right) \right)$,

Let $S_{x}^{'}=\exp\left( -\frac{w_{i}}{N_{i}}\left( R_{i}+M_{i}-D_{i} \right) \right),$ $\log\left( S_{x}^{'} \right)=-\frac{w_{i}}{N_{i}}\left( R_{i}+M_{i}-D_{i} \right)$, and $p_{xi}=\frac{R_{i}}{R_{i}+M_{i}-D_{i}}$，then

Var ($\log S_{x}^{'})=\left\{ \begin{aligned} {w_{i}}^{2}\frac{p_{xi}\left( 1-p_{xi} \right)}{N_{i}}, (Binomial) \\ {w_{i}}^{2}\frac{p_{xi}}{N_{i}}, (Poisson) \end{aligned} \right.$

Let $S_{x}=1-e^{\hat{\theta}},$ according to delta method，then：

$$E\left( S_{x} \right)=\left( 1-\exp\left( -\frac{w_{i}}{N_{i}}\left( R_{i}+M_{i}-D_{i} \right) \right) \right), \left( Binomial \& Poisson \right) (6)$$

$Var\left( S_{x} \right)={(e^{\hat{\theta}})}^{2}\times Var\left( e^{\hat{\theta}} \right)=\left\{ \begin{aligned} {(S_{x}^{'})}^{2}\times{w_{i}}^{2}\frac{p_{xi}\left( 1-p_{xi} \right)}{N_{i}}, (Binomial) \\ {(S_{x}^{'})}^{2}\times{w_{i}}^{2}\frac{p_{xi}}{N_{i}}, (Poisson) \end{aligned} \right.$ (7)

**Frontiers analysis**

To evaluate the relationship between disease burden and socio-demographic development, we employed frontier analysis as a quantitative approach to identify the lowest achievable lifetime incidence and mortality risk based on the level of development measured by the Socio-demographic Index (SDI). Frontier analysis determines the minimum lifetime risk that each country or region could attain given its SDI. The distance from the frontier, referred to as the effective difference, quantifies the gap between the observed and the theoretically achievable risk. A larger effective difference suggests the presence of unrealized opportunities for improvement (i.e., reduction in lifetime risk of BPH) that could be achieved based on the country or region’s position along the development spectrum.

We applied Data Envelopment Analysis (DEA) using the Free Disposal Hull (FDH) method to construct a nonlinear frontier, utilizing data from 1990 to 2021 to generate SDI-stratified BPH frontiers ^[3,4]^. To account for uncertainty, we performed 1,000 bootstrap resampling iterations, randomly sampling (with replacement) from all countries and regions across all years. The average lifetime disease risk was then calculated for each SDI level. Subsequently, we employed locally weighted regression (LOESS regression) with a local polynomial degree of 1 and a smoothing span of 0.2 to generate a smoothed frontier curve. To mitigate the influence of outliers, super-efficient countries were excluded from the frontier estimation ^[9]^.

**Reference:**

1. Sasieni P, Shelton J, Ormiston-Smith N, et al. What is the lifetime risk of developing cancer?: The effect of adjusting for multiple primaries. Brit J Cancer 2011;105 (3):460-5

2.Ahmad AS, Ormiston-Smith N, Sasieni PD. Trends in the lifetime risk of developing cancer in Great Britain: comparison of risk for those born from 1930 to 1960. Br J Cancer 2015;112:943-7.

3. Access GBDH, Quality Collaborators. Electronic address cue, Access GBDH, Quality C. Healthcare Access and Quality Index based on mortality from causes amenable to personal health care in 195 countries and territories, 1990-2015: a novel analysis from the Global Burden of Disease Study 2015. Lancet. May 18 2017.

4. Xie Y, Bowe B, Xian H, Balasubramanian S, Al-Aly Z. Rate of Kidney Function Decline and Risk of Hospitalizations in Stage 3A CKD. Clinical journal of the American Society of Nephrology : CJASN. Nov 6 2015;10(11):1946-55.

**Supplementary Figure 1.** Frontier analysis illustrating the dynamic relationship between SDI levels and the lifetime risk of BPH across countries from 1990 to 2021. The black curve represents the frontier, which indicates the minimum achievable risk for a given SDI level based on the fitted model. Each scatter point represents a country's actual lifetime risk of BPH. The colors represent different years.
